# Targeted UHPLC-MS Analysis Reveals Disparate Polyphenol Composition and Concentration in Muscadine Grape Supplements with Proportional Antioxidant Activity

**DOI:** 10.3390/antiox11112117

**Published:** 2022-10-27

**Authors:** Mark C. Chappell, Aja V. Duncan, Ana Clara Melo, Christopher L. Schaich, Nancy T. Pirro, Debra I. Diz, E. Ann Tallant, Patricia E. Gallagher

**Affiliations:** Surgery/Hypertension and Vascular Research, Wake Forest University School of Medicine, Winston-Salem, NC 27101, USA

**Keywords:** keyword muscadine grape supplements, polyphenols, ultra-high pressure liquid chromatography, mass spectroscopy, oxidative stress, superoxide dismutase, catalase, malondialdehyde, 4-hydroxynonenol

## Abstract

Muscadine grape supplements (MGS) with high polyphenol content are a potential therapeutic option to combat oxidative stress; however, the precise identity and concentration of individual phenolics in commercially processed MGSs is not well defined. We probed for 17 phenolic compounds by ultra-high pressure liquid chromatography and mass spectroscopy from distinct lots of four commercially processed MGSs composed of MG seed and/or skin waste products. The total phenolic content (TPC) and antioxidant capacity were highest in a dried water-extract MGS as compared to three ground seed and/or skin products. The TPC was not different between MGS lots from individual companies and remained stable for 3 years without microbial contamination. The extract MGS had the highest concentration of epicatechin, ellagic acid, gallic acid, procyanidin B2, catechin and catechin gallate compared to the other supplements. Only ellagic acid and gallic acid were detected in all four MGSs, while catechin and catechin gallate were below detection in two supplements. Based on gram weight, only the extract MGS prevented the angiotensin II-induced increase in malondialdehyde and 4-hydroxynonenol in rat H9c2 cardiomyocytes as well as upregulated superoxide dismutase and catalase. This study demonstrates that commercial MGSs differ in phenolic composition and concentration, resulting in disparate antioxidant activity.

## 1. Introduction

Grapes (*Vitis vinifera*) are one of the largest fruit crops in the world and are considered a rich source of polyphenolic compounds. In particular, muscadine grapes (MG, *Vitis rotundofilia*) contain a high quantity of antioxidant compounds in relation to other grape varieties. The diversity and concentration of phenolic acids, flavanols, stilbenes and anthocyanins may be of potential benefit for treatment of a variety of pathologies including cancer, diabetes, cognitive decline, and cardiovascular disease [1,2,3,4,5]. MGSs typically contain ground dried skin and seed of the grape, while the pulp is destined for the production of juices, wines and jams. The MG skin and seeds were once considered waste products in the processing of grapes for wine and juices; however, the high concentrations of polyphenols in the seed and skin are the primary basis for their widespread utilization as a nutritional supplement [6,7,8,9].

Although grape seed supplements are considered a potential therapeutic approach to maintain health, there are safety and efficacy concerns [10,11]. The biologically active phenolics in the grapes chosen for supplement manufacture may differ due to cultivar as well as soil type, water availability and weather conditions for that harvest year, leading to variations in the final product. The content and concentration of the polyphenolic compounds in grape supplements are dependent on the ratio of the skin, seed, and pulp included in the supplement [7,8,9,12]. Further, the manufacturing plants do not maintain the same level of quality control, resulting in batch to batch variation, and processing methods may alter the composition and concentration of polyphenolic compounds.

While a number of studies assessed the phenolic content of laboratory preparations of muscadine seeds and skins [7,8,12,13,14], there is limited information on the MGSs commercially available for consumer consumption. In an analysis of 21 commercial grape seed supplements, Villani et al. [15] reported that only five supplements contained gallic acid, a primary phenolic acid in the grape seed and skin. Moreover, six of the supplements were more likely derived from peanut skin and another three from pine bark rather than the actual grape seed based on the profile of gallic acid and the proanthocyanins catechin and epicatechin [15]. However, the Villani study did not specify the type or variety of grape seed of each supplement, nor did it extend their analysis beyond gallic acid and proanthocyanins [15]. While little is known about the composition and potential antioxidant activity of muscadine grape supplements (MGSs) currently on the market, consumers are purchasing and ingesting these products daily, researchers are utilizing MGSs in experimental studies, and more importantly, clinical trials are ongoing using MGSs. The goal of this study was to determine whether there are differences in total phenolic content, antioxidant capacity, concentration of common MG phenolic compounds, and lot variation from four commercially processed MGSs to provide a basis for the use of these products by the general public, laboratory scientists, and clinicians. The MGSs examined include a proprietary supplement (Piedmont Research Development Corporation, PRDC) that undergoes an additional enrichment process and three widely available commercial supplements. The PRDC and Muscadine Naturals Premier Gold (PG) supplements are associated with beneficial effects in patient clinical trials [16,17,18]. The supplements were derived from either the MG seed and skin (Nature’s Pearl (NP), PRDC and Muscadinex (MX)) or skin PG. The study utilized a targeted approach to identify and quantify 17 endogenous polyphenolic compounds by ultra-high pressure liquid chromatography–mass spectroscopy (UHPLC-MS). Antioxidant activity of the individual MGSs was assessed in cardiomyocytes to determine whether phenolic concentration and content affected antioxidant activity.

## 2. Materials and Methods

### 2.1. Chemicals

The phenolic standards used in the study were of the highest quality available and include catechin hydrate, resveratrol, p-coumaric, gallic acid (certified reference material), ferulic acid (European Pharmacopeia Reference standard), epicatechin (primary pharmaceutical reference standard), quercetin, caffeic acid, ellagic acid, procyanidin B2 (analytical standard), catechin gallate, epigallocatechin, gallocatechin, gallocatechin gallate, myricetin (analytical standard), and kaempferol (Sigma-Aldrich, St. Louis, MO, USA). MS grade formic acid, Folin–Ciocalteu phenol reagent and sodium carbonate were also purchased from Sigma-Aldrich. The isotopic catechin (2, 3, 4-^13^C3) reference standard was obtained from Cambridge Isotope Laboratories Inc. (Andover, MA, USA). The LCMS grade water and methanol were purchased from Fisher Scientific Co. (Santa Clara, CA, USA).

### 2.2. Commercially Processed MGSs

NP MGS (prepared from skin and seed) was purchased from Nature’s Pearl (Advance, NC, USA), and lots #50-H (NP-1), #119-J (NP-2), and #210-J (NP-3) were analyzed. PG supplement (prepared from MG skin) was purchased from Muscadine Naturals, Inc. (Clemmons, NC, USA), and lots #1503483 (PG-1), #1604240 (PG-2), and 1,709,166 (PG-3) were used for analysis. MX supplement (prepared from skin and seed) was purchased from Muscadinex Inc. (Pine Level, NC, USA), and lots #1026505 (MX-1), #1106622 (MX-2) and #1116516 (MX-3) were analyzed. The PRDC proprietary (PRO) supplement is a dried water extract of MG skin and seed purchased from Piedmont Research Development Corporation (Advance, NC, USA); lots #013-1 (PRO-1), #086-1 (PRO-2) and #219-1 (PRO-3) were analyzed in this study. Microbial analysis (total aerobic microbial content, total yeast and mold content, detection of *Escherichia coli*, *Pseudomonas aeruginosa*, *Salmonella*, and *Staphylococcus aureus*), heavy metal content (arsenic, cadmium, lead, and mercury) and pesticide concentration was performed by American Testing Lab (San Diego, CA, USA).

### 2.3. Extraction of Phenolic Compounds

MGSs (10 mg each) were diluted 1:100 in 70% methanol/1.0% formic acid and pulverized in a Qiagen TissueLyser (Germantown, MD, USA) with 2 metal beads for 5 min at a speed of 30 Hz. The homogenates were rotated for 60 min at room temperature and centrifuged at 100,000× *g* at 4 °C in an ultracentrifuge (Beckman-Coulter Life Sciences; Indianapolis, IN, USA). The supernatant samples were quantified for total phenolic content by the Folin–Ciocalteu method and individual phenolics by targeted UHPLC-MS analysis as described below. For the UHPLC-MS analysis, 10 µg of isotopically labeled catechin was added as an internal standard before the sample extraction.

### 2.4. Total Phenolic Content (TPC)

TPC for each extract was quantified by the Folin–Ciocalteu (FC) assay as described by Singleton and Rossi with modification [19]. Briefly, 0.1 mL of supernatant (diluted 1:10) from each sample was mixed with 0.5 mL of the FC reagent (diluted 1:10) and reacted for 1 min at room temperature. Subsequently, 0.4 mL of 7.5% sodium carbonate was added to the mixture and incubated for an additional 40 min at 37 °C. Three determinations were made per lot. The UV absorbance (750 nm) was measured in a BioRad Benchmark Plus plate reader (Hercules, CA, USA). TPC was expressed as mg/g dry weight of the initial sample using gallic acid as the phenolic standard (10–100 µg/mL).

### 2.5. Quantification of Antioxidant Capacity

The supplements were resuspended in deionized water, stirred for 2 h and filtered through Whatman #4 filter paper under vacuum. The antioxidant activity of each preparation was measured by 2,2-diphenylpicrylhydrazyl (DPPH) radical scavenging activity. The samples were mixed with 200 μM DPPH dissolved in methanol and incubated at room temperature for 30 min in the dark. Trolox served as a standard (10–500 μM). The absorbance of triplicate samples per lot was measured at 515 nm, and the antioxidant capacity was expressed as μmol Trolox equivalent/g dry material.

### 2.6. UHPLC-MS Analysis

Chromatographic separation of the phenolic standards and extracts from the 3 different lots of the 4 supplements was performed by UHPLC-MS (Nexera X2- LC-MS 2020, Shimadzu North America, Columbia, MD, USA) with a 2.1 × 100 mm reverse phase column (Kinetex Biphenyl; Phenomenex Inc, Torrance, CA, USA) at a flow rate of 0.45 mL/min and temperature of 55 °C. The mobile phases consisted of 0.1% formic acid (A) and 100% methanol/0.1% formic acid (B). Separation of the phenolic compounds was achieved with the following gradient: 15–70% B (2.8 min); 70–90% B (0.1 min) and 90% B isocratic (0.3 min). The extracted samples were diluted to the starting chromatographic conditions of 15% B and filtered through a 0.45 µM filter vial (ChromTech; Minneapolis, MN, USA); a 0.01 mL sample volume was injected on the UHPLC. Following the chromatographic separation, the fractions were analyzed with a Shimadzu 2020 MS with an ESI source in the negative mode. The scan range was 50–500 *m*/*z*, and scan speed was 7500 u/sec. The MS conditions were as follows: nebulizing gas flow rate 1.5 L/min, drying gas flow 15.0 L/min, interface voltage 4.5 kV, detector voltage 1.0 kV, interface temperature 350 °C, DL temperature 250 °C, and a heat block temperature of 400 °C. For quantification of the phenolic content, stock solutions of phenolic standards were prepared in 100% methanol. Each supplement lot was assayed three times; the mean value was calculated for each phenolic among the different lots and then statistically compared to the other supplements. A five-point calibration curve of 2, 5, 25, 50 and 100 ng for the gallic acid standard was generated with 0.01 mL sample volume injections, and the data fit to a simple linear regression curve (y − 0.2786 * X − 0.2956, *r* = 0.9975) with the least quantifiable unit of detection (LOQ) of 0.2 μg/mg (0.2 mg/gm) of supplement.

### 2.7. Quantification of Antioxidant Markers and Enzymes

The rat myocardial cell line H9c2 (CRL-1446) was purchased from the American Type Culture Collection (Manassas, VA, USA). H9c2 cells were maintained in Dulbecco’s Modified Eagle’s Medium (DMEM) supplemented with 100 µg/mL penicillin, 100 units/mL streptomycin, 15 mM HEPES, 2 mM L-glutamine and 10% fetal bovine serum (FBS). The cultures were maintained at 37 °C and 5% CO_2_.

H9c2 cells growing in media with 0.5% serum were incubated at 37°C with 100 nM angiotensin II (Ang II), 20 μg dry weight of MGSs or the combination of Ang II and a supplement. Angiotensin II induces oxidative stress in the heart under pathological conditions, thus providing a clinically relevant oxidant for these cell culture studies [20,21]. The Ang II and MGES dose was based on previous studies [22,23,24]. Proliferation was monitored with the IncuCyte ZOOM System (Sartorious, Gottingen, Germany), with images collected every 2 h, as a measure of cell viability.

After 24 h, the cardiomyocytes were homogenized in 10 mM Tris-hydrochloric acid (Tris-HCl; pH 7.4) at 4 °C, and the homogenate was subjected to centrifugation at 4000× *g* for 30 min at 4 °C. The resultant supernatant was collected, and protein concentrations were determined using the protein Bio-Rad assay. The activities of endogenous cardiomyocyte malondialdehyde (MDA), 4-hydroxynonenol (4-HNE), catalase and superoxide dismutase1 (SOD1) were measured using assay kits from Cayman Chemical (Ann Arbor, MI, USA) according to the manufacturer’s protocol. Four determinations of each antioxidant or enzyme were made. The MDA and enzyme activity were normalized to the total protein concentration and expressed as Units (U)/mg protein. 4-HNE was expressed as μmol/mg protein.

### 2.8. Data and Statistical Analysis

All data are expressed as the mean ± SEM. Statistical comparisons were shown using compact letter display with only significant comparisons (*p* < 0.05) labeled. One-way ANOVA followed by Tukey’s post hoc test was performed for the total soluble phenolic content, polyphenol concentrations among the commercial supplements, and the antioxidant activity with statistical significance at *p* < 0.05. Correlation analysis was performed for the mean values of total phenolic content and the individual phenolic concentrations among the four MGSs. A repeated measures multivariate ANOVA (MANOVA) was used to determine whether TPC in different MGS lots was similar within respective MG supplements and remained stable over time. The analysis included supplement, lot, and year terms and their 3-way interaction. All univariate analyses were performed and all graphs constructed using GraphPad Prism 9.01 for Windows (La Jolla, CA, USA). Multivariant analyses were conducted in R 4.1.2 (R Foundation, 2021).

## 3. Results

### 3.1. Total Phenolic Content (TPC) and Antioxidant Capacity of MGSs

**Figure 1:**
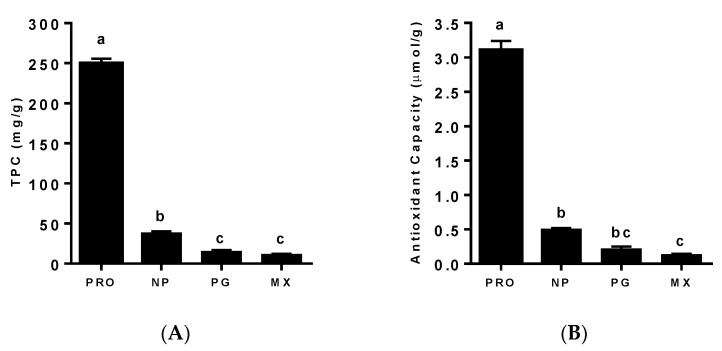
**Total soluble phenolic content and antioxidant capacity for the four MGSs.** (**A**) Total phenolic content (TPC) was quantified by the Folin–Ciocalteu assay and expressed as mg/g of MGS. (**B**) Antioxidant capacity was quantified by DPPH radical scavenging activity using Trolox as a control. The MGSs analyzed were PRDC, lots 1-3 (PRO 1-3); Nature’s Pearl, lots 1-3 (NP 1-3); Premier Gold, lots 1-2 (PG 1-3); and Muscadinex, lots 1-2 (MX 1-3). Data are the mean ± SEM; *n* = 3 determinations per lot. Statistical comparisons are labeled using compact letter display with only significant differences (*p* < 0.05) shown.

The TPC of the MGSs from three different lots of the four suppliers was quantified. As shown in Figure 1A, the TPC was significantly higher in the PRO MGS as compared to the NP (PRO—249 ± 6.6 vs. NP—38.5 ± 1.6; 6.5-fold), PG (PRO—249 ± 6.6 vs. PG—15.4 ± 1.2; 16-fold) and MX (PRO—249 ± 6.6 vs. MX—11.9 ± 0.2; 21-fold) supplements. The TPC in the NP supplement was approximately two-fold higher than that of PG or MX powders (Figure 1A).

Similar to the TPC, the antioxidant capacity of the PRDC supplement was markedly higher than the Nature’s Pearl (PRO—3.09 ± 0.14 vs. NP—0.5 ± 0.01), Premier Gold (PRO—3.09 ± 0.14 vs. PG—0.22 ± 0.03) or Muscadinex (PRO—3.09 ± 0.14 vs. MX—0.13 ± 0.01) supplements (Figure 1B). While the antioxidant capacity of the Premier Gold powder did not differ from the Nature’s Pearl or Muscadinex supplements, the Muscadinex samples had a two-fold reduction in antioxidant capacity as compared to the Nature’s Pearl supplement.

**Figure 2:**
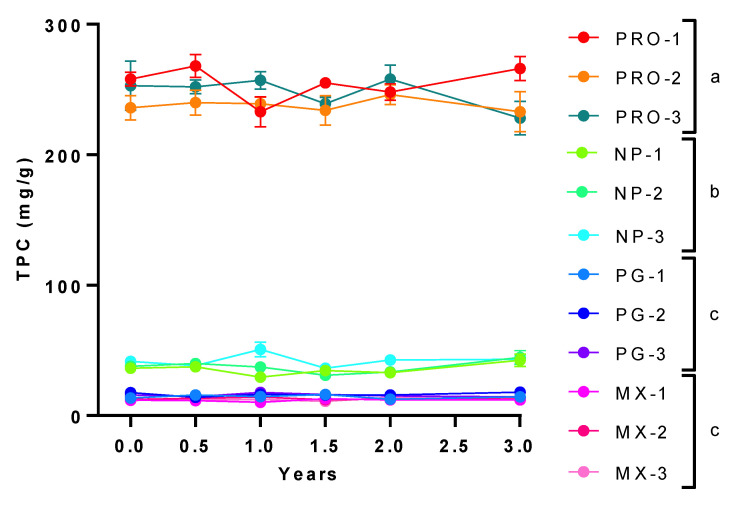
**Total Phenolic Content of MGS Lots.** Total phenolic concentration (TPC) from 3 different lots of the 4 commercially processed muscadine grape supplements PRDC (PRO), Nature’s Pearl (NP), Premier Gold (PG) and Muscadinex (MX). All supplements were extracted and TPC expressed as mg/g sample. Data are mean ± SEM of 3 determinations from each lot. Statistical comparisons are labeled using compact letter display with only significant differences (*p* < 0.05) shown.

Although we noted marked difference in total phenolic content between each lot of PRO and the lots of the other supplements, there was no significant variation in overall phenolic content within the different lots of each supplement (Figure 2). In addition, the TPC in the different lot samples of all four supplements remained stable for three years (Figure 2), and no evidence of antimicrobial contamination was observed after storage at room temperature for this period. Specifically, the aerobic microbial count and total yeast and mold count were consistently <10 colony-forming units per gram. *Escherichia coli*, *Staphylococcus aureus*, *Salmonella*, and *Pseudomonas aeruginosa* were undetectable. Pesticide levels conformed to the United States Pharmacopeia requirements at baseline. The heavy metals arsenic, cadmium, lead and mercury were below 0.01 parts per million.

### 3.2. UHPLC-MS of Commercially Processed MGSs

**Figure 3:**
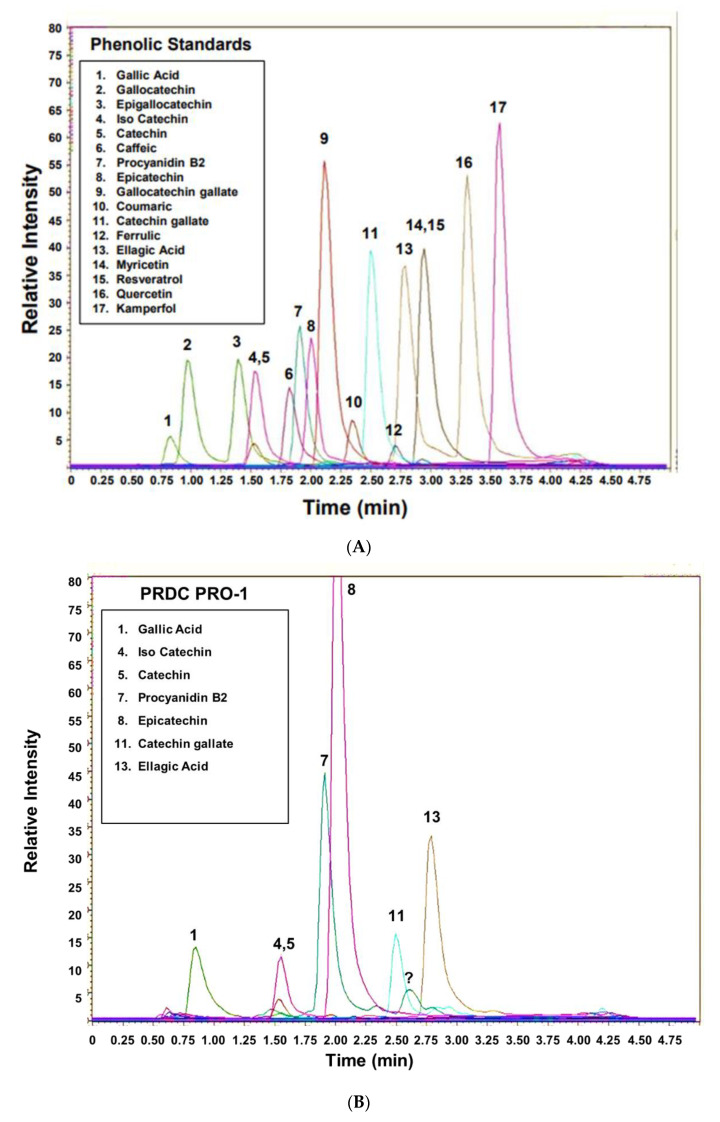

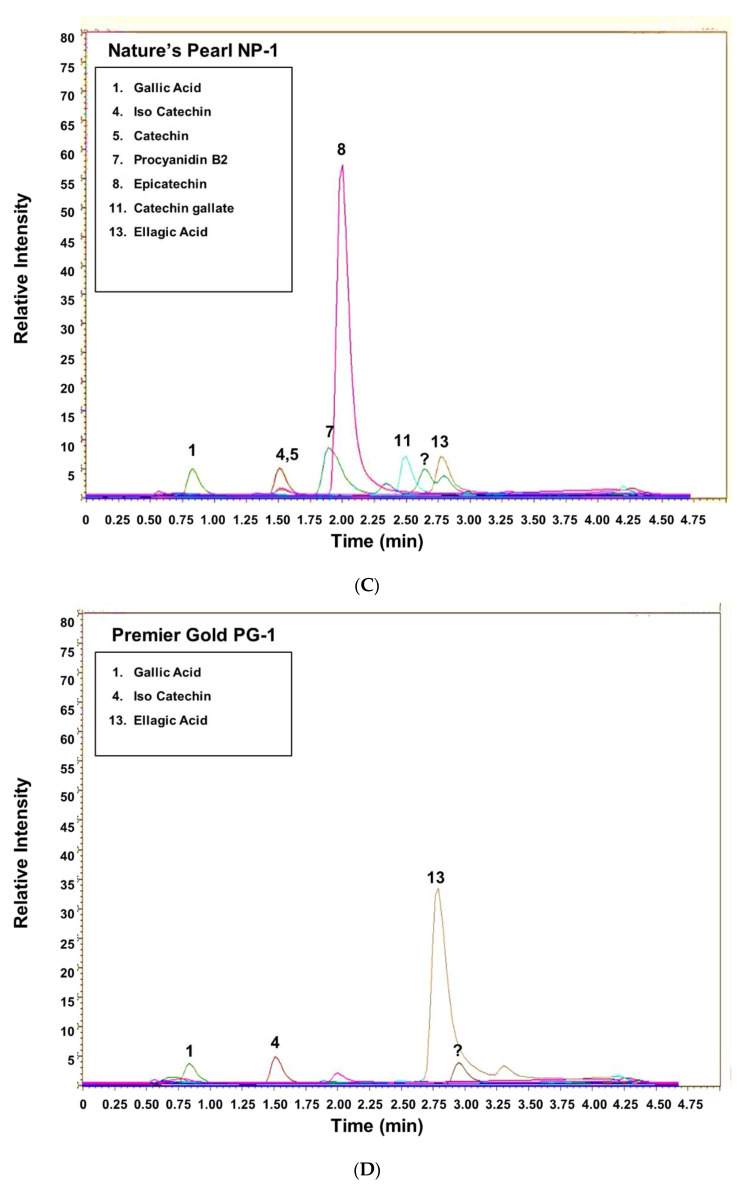

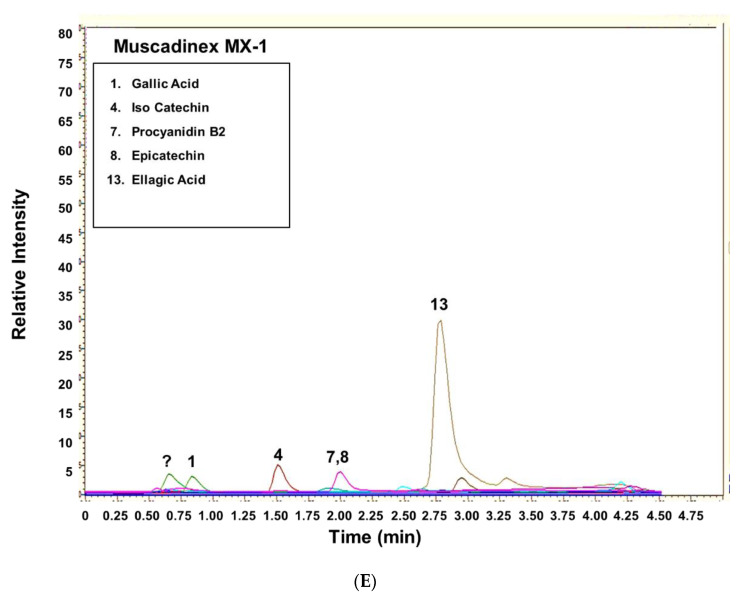
**Representative chromatograms of MGSs.** (**A**) UHPLC-MS of polyphenolic standards. Extracts (0.01 mL) of the four commercially processed MGSs were analyzed by UHPLC-MS. (**B**) Chromatogram of the PRDC lot (PRO-1) MGS extract reveals major peaks for epicatechin, gallic acid and procyanidin B2. (**C**) Chromatogram of Nature’s Pearl MGS lot (NP-1) shows a major peak of epicatechin. (**D**) Chromatogram of Premier Gold (PG) MGS lot (PG-1) reveals peaks of ellagic and gallic acid. (**E**) Chromatogram of Muscadinex (MX) MGS lot (MX-1) reveals peaks of ellagic and gallic acid, as well as epicatechin.

The individual phenolics from each extracted supplement were identified by their retention times and mass/charge ratio (*m*/*z*^−1^) by UHPLC-MS; isotopically labeled catechin was added to each supplement prior to extraction and served as an internal standard. As shown in Figure 3A, the UHPLC-MS chromatograph revealed that the majority of the 17 phenolic standards are well-separated within the 4 min span of the separation conditions employed in this analysis (Supplementary Table S1). Catechin and its isotopically labeled analog co-eluted, as well as myricetin and resveratrol; however, these compounds were resolved by their different molecular mass (Figure 3A, Supplementary Table S1). A solid phase enrichment step of the initial extract was not included over concern with potential differences in the recovery of the various phenolic compounds. A 100,000× *g* centrifugation step was employed to clarify the extract. In this approach, the sample injection volume of the extract was also limited to 0.01 mL to obviate any matrix effects on the MS detection of the individual phenolics. Samples below the LOQ (<0.2 μg/mg) were assigned a non-detectable or zero value.

Figure 3B–E show representative UHPLC-MS chromatographs from an identical sample volume (0.01 mL) of the different MGSs extracts. UHLPC-MS of the PRDC proprietary supplement PRO-1 revealed peaks corresponding to epicatechin, catechin, procyanidin B2, catechin gallate, ellagic and gallic acid; however, peak signals corresponding to myricetin, resveratrol or kaempferol were not detected (Figure 3B). Nature’s Pearl NP-1 exhibited peaks that corresponded to epicatechin, catechin, procyanidin B2, catechin gallate, ellagic acid and gallic acid (Figure 3C). Prominent peaks of gallic and ellagic acid were also detected in the extract of the Premier Gold PG-1 (Figure 3D). The phenolic profile of the Muscadinex MX-1 skin/seed supplement contained peaks of ellagic acid, gallic acid, epicatechin and procyanidin B2 (Figure 3E).

### 3.3. Polyphenol Content in Commercial MGSs

**Figure 4:**
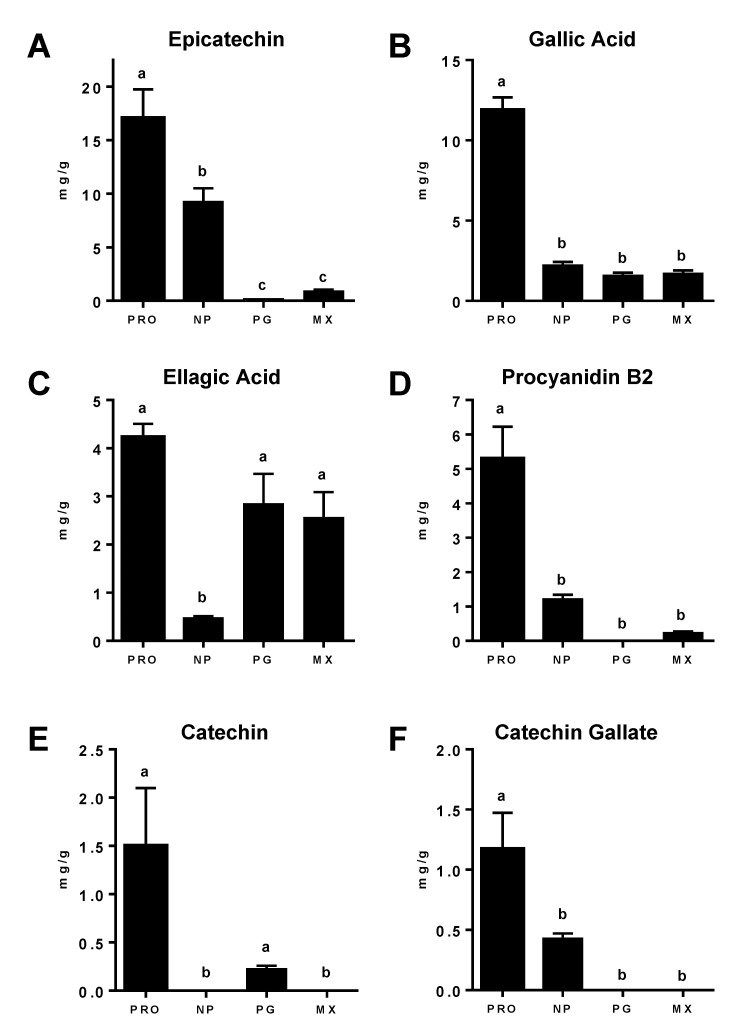
**Individual phenolic content of the MGSs.** Individual phenolics, epicatechin (**A**), gallic acid (**B**), ellagic acid (**C**), procyanidin B2 (**D**), catechin (**E**), and catechin gallate (**F**) of three different lots of the PRDC (PRO), Nature’s Pearl (NP), Premier Gold (PG), or Muscadinex (MX) were analyzed for content by UHPLC-MS and expressed as mg/g. Data are the mean ± SEM of 3 different lots with 3 determinations per lot. Statistical comparisons are labeled using compact letter display with only significant differences (*p* < 0.05) shown.

As shown in Figure 4, the PRDC proprietary supplement contained the highest content of epicatechin, ellagic acid, gallic acid, procyanidin B2, catechin and catechin gallate as compared to the other supplements based on the initial gram weight of each supplement. The Nature’s Pearl NP supplement contained the second highest amount of epicatechin with lower amounts of ellagic acid, catechin gallate and procyanidin B2; the gallic acid content was comparable to that in the Premier Gold and Muscadinex MGSs (Figure 4). The levels of epicatechin, procyanidin B2, catechin and catechin gallate in the Premier Gold MG skin supplement were below the detection limit of the current assay. The Muscadinex MX skin/seed supplement contained comparable levels of ellagic acid and gallic acid compared to Premier Gold supplement, but a higher content of both epicatechin and procyanidin B2; catechin and catechin gallate were below the detection limit for the MX extracts (Figure 4).

**Figure 5:**
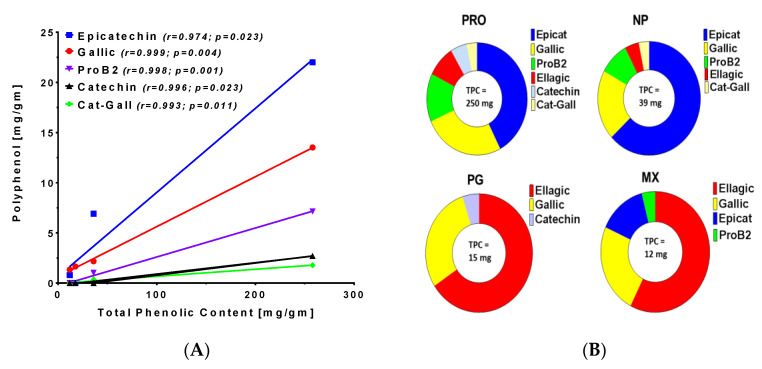
**Analysis of MGS polyphenol content.** (**A**) Fractional expression of polyphenols in the muscadine grape supplements. (**B**) Correlation of the total phenolic content to individual polyphenols in the muscadine grape supplements PRDC (PRO), Nature’s Pearl (NP), Premier Gold (PG) and Muscadinex (MX). Epicat (epicatechin); Gallic (gallic acid); ProB2 (procyanidin B2); Cat-Gall (catechin gallate).

The fractional percentages for each individual phenolic identified by UHPLC-MS were calculated to convey the different phenolic profiles among the four supplements (Figure 5A). Epicatechin and gallic acid were the major phenolics in PRDC and Nature’s Pearl supplements while ellagic acid and gallic acid were the primary components of the Premier Gold and Muscadinex MG products. A correlation analysis of total phenolic content and the individual polyphenols in the four supplements was performed. This analysis revealed a high degree of linearity (*r* values) and significance (*p* values) for epitcatechin, gallic acid, procyanidin B2, catechin, and catechin gallate (Figure 5B). In contrast, levels of ellagic acid correlated poorly with the total phenolic content in the four supplements.

### 3.4. Effect of Commercially Prepared MGSs on Oxidative Stress

**Figure 6:**
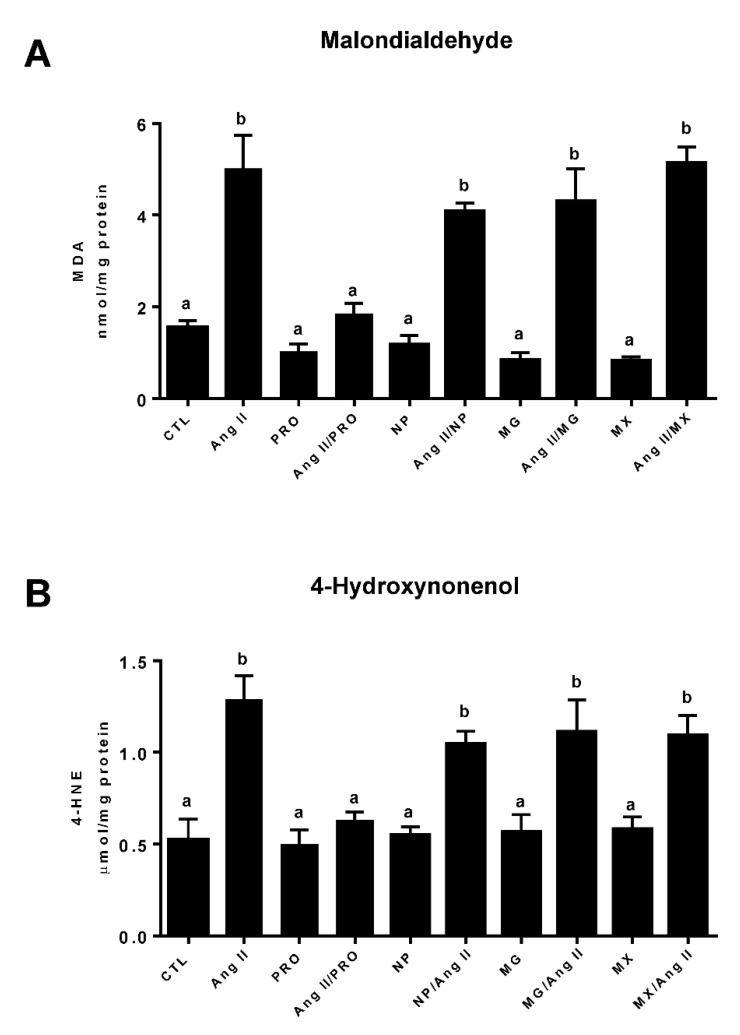
**PRDC MGS ameliorated the Ang II-mediated increase in markers of oxidative stress in rat cardiomyocytes.** (**A**) Quantification of MDA in homogenates of rat H9c2 cells. (**B**) Quantification of 4-HNE in homogenates of rat H9c2 cells. Values are mean ± SEM; *n* = 4 per group. Statistical comparisons are labeled using compact letter display with only significant differences (*p* < 0.05) shown. Muscadine grape supplements PRDC (PRO), Nature’s Pearl (NP), Premier Gold (PG) and Muscadinex (MX).

MDA and 4-HNE were quantified in rat H9c2 cardiomyocytes to determine whether the commercially produced MGSs attenuate oxidative stress caused by Ang II. Oxidative stress plays a crucial role in the development of cardiac damage resulting from hypertension and elevated Ang II. As shown in Figure 6A, Ang II treatment of H9c2 myocytes increased the concentration of MDA, a reactive aldehyde product of lipid peroxidation, by more than three-fold as compared to control (CTL) cells (CTL—1.54 ± 0.16 vs. Ang II—5.01 ± 0.83). Co-incubation of rat cardiomyocytes with 20 μg dry weight/mL of the PRO MGS effectively prevented the Ang II-induced increase in MDA (Ang II—5.01 ± 0.83 vs. Ang II/PRO 1.85 ± 0.23). Conversely, 20 μg/mL of NP, PG, or MX supplements did not block the induction of MDA by Ang II (Figure 6A). The dry weight dose of 20 μg/mL used is equivalent to approximately 5.0 μg total phenolics/mL of PRO, 0.75 μg total phenolics of NP, 0.3 μg total phenolics of PG and 0.2 μg total phenolics of MX. Addition of the MGSs alone to H9c2 cells had no effect on MDA concentration. The PRO MGS (20 mg/mL) also prevented the Ang II-induced increase in 4-HNE (Ang II—1.28 ± 0.13 vs. Ang II/PRO 0.63 ± 0.04), as shown in Figure 6B, while co-treatment with NP, PG, or MX supplements did not attenuate the Ang II increase in cardiomyocyte 4-HNE. Incubation of H9c2 cells with the MGSs alone had no effect on 4-HNE concentrations. Further, no statistically significant change in cell proliferation was observed between the control and treatment groups, demonstrating stable cell viability for the 24 h period.

### 3.5. Effect of Commercially Prepared MGSs on Antioxidant Enzymes

**Figure 7:**
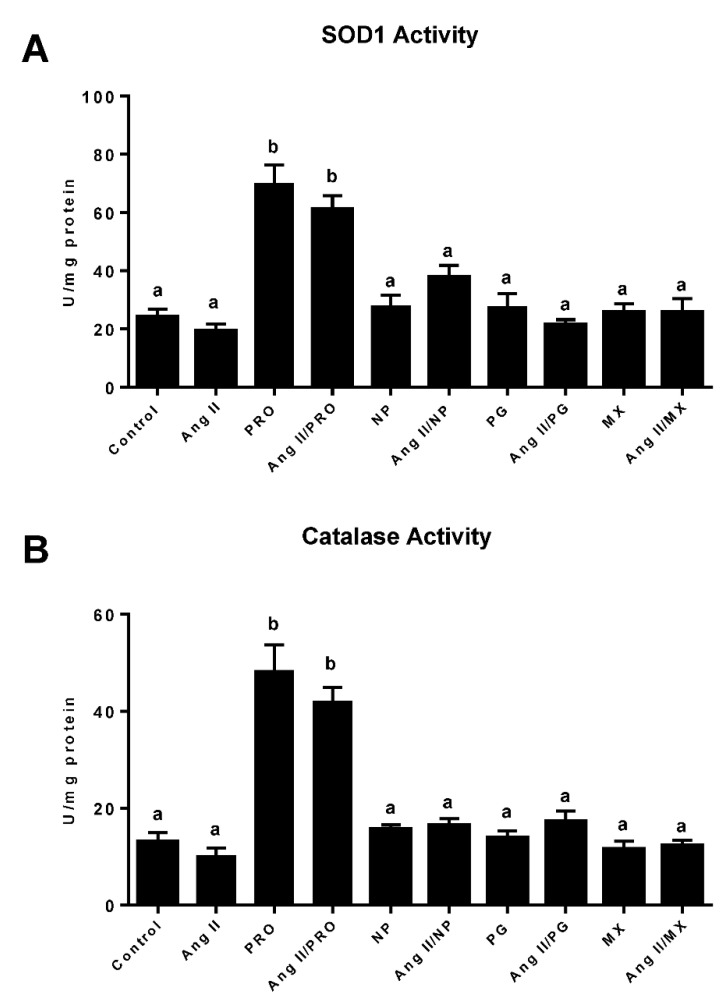
**PRDC MGS enhanced the antioxidant activity in rat cardiomyocytes.** (**A**) Quantification of SOD1 activity in homogenates of rat H9c2 cells. (**B**) Quantification of catalase activity in homogenates of rat H9c2 cells. Statistical comparisons are labeled using compact letter display with only statistically significant differences (*p* < 0.05) shown. Muscadine grape supplements PRDC (PRO), Nature’s Pearl (NP), Premier Gold (PG) and Muscadinex (MX).

SOD1, which converts the superoxide radical to hydrogen peroxide, and catalase, which catalyzes the decomposition of hydrogen peroxide to oxygen and water, are two key antioxidant enzymes critical for the prevention of cardiac damage by oxidative stress. Ang II was incubated with rat H9c2 myocytes to induce reactive oxygen species production to determine whether the commercially produced MGSs increased the protective enzymes SOD1 and catalase. Treatment of cardiomyocytes with PRO MGS (20 μg dry weight/mL) with or without Ang II resulted in a marked increase in cardiac SOD1 activity as compared to control or Ang II cell extracts (*p* < 0.0001 PRO MGS compared to CTL or Ang II; *p* > 0.0001 Ang II + PRO compared to CTL or Ang II alone) (Figure 7A). Incubation with Ang II only had no significant effect on myocyte SOD1 activity. Incubation of H9c2 cells with 20 μg dry weight/mL MGSs from Nature’s Pearl, Premier Gold, or Muscadinex with or without Ang II did not upregulate SOD1 activity. The dry weight dose of 20 μg/mL is equivalent to approximately 5.0 μg total phenolics/mL of PRO, 0.75 μg total phenolics of NP, 0.3 μg total phenolics of PG and 0.2 μg total phenolics of MX. The PDRC MGS alone or in combination with Ang II also resulted in a significant increase in cardiomyocyte catalase activity in rat cardiomyocytes as compared to control or Ang II extracts (Figure 7B; *p* < 0.0001 PRO compared to CTL or Ang II; *p* < 0.0001 PRO + Ang II compared to CTL or Ang II alone). No increase in catalase activity was observed after incubation of H9c2 cells with Ang II alone or 20 μg/mL MGSs from Nature’s Pearl, Premier Gold, or Muscadinex with or without Ang II. In addition, no statistically significant change in cell viability was observed between the control and treatment groups during the incubation time, as demonstrated by consistent proliferation.

## 4. Discussion

This study is the first to show by targeted analysis distinct differences in TPC, antioxidant capacity, as well as profile and concentration of individual phenolic compounds of commercially processed MGSs. Further, these differences were reflected in the disparate biological activity of the MGSs as assessed by oxidant byproducts and antioxidant enzyme activity.

Only ellagic acid and gallic acid were detected in all four MG supplements. The levels of ellagic acid were approximately 40% higher in PRO than the PG and MX (although this did not reach statistical significance) and 10-fold higher than that found in NP. Decades of study show that ellagic acid has protective effects against a number of pathologies, including cancer, diabetes, cardiovascular disorders, and neurogenerative conditions, due to reductions in inflammation, proliferation, and oxidation [25,26]. Gallic acid content was similar for NP, PG and MX, approximately 2 mg/g, but was markedly higher in the PRO supplement (>12 mg/g). In comparison, You et al. reported similar levels of gallic acid and ellagic acid (0.2–0.3 mg/g) in acetic acid/methanol extracts of the MG seed in which the extracts were concentrated by a solid phase step (SepPak C18) prior to HPLC-MS analysis [14]. These investigators also found that the content of ellagic acid was 10-fold higher than gallic acid (0.3 vs. 0.025 mg/g) in extracts of the MG skin [14]. Of particular interest to our study, Villani et al. [15] reported detectable levels of gallic acid in only 5 of 21 commercial grape seed supplements that ranged from 11 to 102 mg/g in content. Gallic acid exhibits beneficial effects through anti-oxidant, anti-proliferative, and anti-inflammatory actions [27,28,29,30].

We identified procyanidin B2 and the monomer epicatechin in the PRO, NP and MX supplements, but both compounds were absent or below detection in the PG MGS. The PRO MGS also contained significantly higher levels of procyanidin B2 (>7 mg/g) than NP (1.2 mg/g) or MX (0.25 mg/g). Several reports demonstrate beneficial effects of a proanthocyanin extract from grape seed on nephropathy, inflammation and oxidative stress in experimental diabetes [31,32,33,34]. The proanthocyanin extract ameliorated amyloid accumulation and cognitive dysfunction in a pre-clinical model of Alzheimer’s disease [35,36,37]. The analysis of the PRO supplement had particularly high levels (>5 mg/g) of gallic acid, procyanidin B2 and epicatechin. Catechin–gallate was detected only in the PRO and NP supplements; however, the level was five-fold higher in the PRO MGS. The epicatechin content was highest among all polyphenols identified in the PRO supplement (approximately 17 mg/g); this was 2- and 20-fold higher than that found in NP and MX, respectively. Both animal and patient studies show that epicatechin protects against the development of diabetes and cardiovascular diseases, including hypertension, obesity, dyslipidemia, and insulin resistance [38,39]. It is likely that the greater content and variety of these potentially therapeutic polyphenols in the PRO supplement reflect an effective enrichment of the soluble phenolic compounds of the MG seeds and skin, as well as the MG variety and growing conditions [15,40].

The failure to detect other phenolic compounds such as quercetin, kaempferol and resveratrol that garnered intense biomedical interest is likely due to their lower content in the MG seed and skin as compared to other phenolics. You et al. reported quercetin, kaempferol and resveratrol levels of 0.02, 0.007 and 0.03 mg/g, respectively, in the MG seed [13,14]. In the MG skin, quercetin and kaempferol levels were lower at 0.007 and 0.001 mg/g, respectively, and resveratrol was not detected [13,14]. The content of these compounds is well below the detection limit (0.2 mg/g) of the current method. Furthermore, You et al. extracted 20 g of MG material as opposed to 0.01 g of MG supplements in our study as well as utilized solid phase C18 columns to concentrate their samples [13,14]. Moreover, it is unclear whether internal standards were utilized in the You et al. study to account for recovery of phenolic compounds during the extraction process of the MG seed and skin, which may also contribute to differences in the content of phenolics between the two studies.

PRO contained the highest levels of ellagic acid among the four supplements, and this polyphenol also exhibits anti-inflammatory and anti-fibrotic actions in cardiac and renal models of injury [25,26,41]. The microbiome-dependent metabolism of ellagic acid yields urolithins, which have renoprotective effects, and the biotransformation to urolithins may represent the endogenous biologically active form of ellagic acid [42,43]. Our recent findings show that urolithin A abolished both transforming growth factor-β and epidermal growth factor (EGF)-stimulated release of the pro-fibrotic compound plasminogen activator inhibitory-1 (PAI-1) in renal epithelial cells that may reflect the inhibition of EGF receptor autophosphorylation [44]. Taken together, our study suggests that the higher content of phenolic compounds either individually or together may contribute to the potential health benefits of the PRO supplement.

Although epicatechin, gallic acid, procyanidin B2, catechin, and catechin gallate highly correlated with overall phenolic content, the concentrations of the identified phenolics do not completely account for the total phenolic values, particularly in the PRDC supplement. It is likely that other compounds contribute to the overall phenolic content in these supplements, and further analysis is warranted to establish their identity and concentration.

A major concern for the use of MGSs as efficacious therapeutics is the potential for variability in the products from year to year, due to soil type, environmental conditions of the growing season, the inclusion of different grape cultivars, the ratio of the skin, seed, and pulp included in the supplement, and changes in the processing methods [10,11]. Surprisingly, there was no significant difference in TPC between lots of the commercially prepared MGSs from a specific company. The lots were purchased over a three-year span to ensure that the grapes were from different harvest years. In addition, the TPC in the four commercially prepared MGSs was unchanged after three years of storage, and no evidence of increased antimicrobial contamination was observed after storage at room temperature for this period. These are important characteristics for use as a therapeutic or a supplement. The presence of pesticide and heavy metals levels were limited, as grape vines may absorb potentially harmful components from the soil, indicating that a reputable grape source is essential. Our study suggests that companies can make a safe and stable MGS that does not differ from batch to batch. However, there are considerable differences in the phenolic composition and content of the MGSs from one company to another. This is a major concern when considering the use of MGS as a therapeutic or for consumer consumption as a preventive agent.

While endogenous antioxidants and antioxidant enzymes maintain the redox balance in the heart, pathological conditions, such as elevated Ang II leading to hypertension, can overload the system with reactive oxygen species, resulting in cardiac damage. We show for the first time that an MGS PRO from PRDC effectively prevented an increase in the oxidative products MDA and 4-HNE in cardiomyocytes treated with Ang II. MDA and 4-HNE, end-products of lipid peroxidation, increase membrane permeability and alter membrane-bound enzymes/ion channels contributing to impaired cardiac function. In addition, incubation of rat cardiomyocytes with MGS PRO increased endogenous SOD1 and catalase activity, suggesting enhanced scavenging of toxic free radicals. Both SOD1 and catalase are regulated by redox-sensitive transcription factors, by post-transcriptional regulation (mRNA stability), by post-translational modification and by epigenetic alterations. The precise mechanism(s) for the regulation of these antioxidant enzymes by PRO is under current investigation. MGSs from NP, MG, or MX did not ameliorate the increase in MDA and 4-HNE or enhance SOD1 or catalase activity in the H9c2 cells under the assay conditions employed. As shown in our study, the PRO MGS, a dried water extract of ground muscadine skins and seeds, had higher TPC, antioxidant capacity, and concentration of phenolics as compared to the NP, MG, and MX MGSs, likely leading to the observed enhanced biological activity. The TPC of the PRO MGS is over 6-fold higher than NP, over 16-fold higher than PG and 25-fold higher than MX. With increased phenolic concentration and/or incubation time, a similar decrease in oxidant byproducts and an increase in antioxidant enzymes might be observed with the NP, MG, and MX supplements. While further study is warranted, our results indicate that the MGS phenolic content and concentration are critical when considering these supplements for medicinal use. Importantly, our study also illustrates that total phenolic content rather than dry weight of MG powders is a more biologically relevant measure for both bench research and clinical studies considering the variability of MG supplements or laboratory preparations.

## 5. Conclusions

This study shows that in a targeted analysis of four commercially processed MGSs, the TPC, antioxidant capacity, profile and concentration of individual phenolic compounds, and antioxidant activity differ significantly. The variability in individual phenolics reflected marked disparities in the overall phenolic content, antioxidant capacity, and biological activity of the MGSs. The current study emphasizes the need for a more complete analysis of phenolic compounds in MGSs and indicates that consumption of MGSs from different commercial suppliers may not yield the expected or comparable intake of polyphenolic compounds or concentration. Although the phenolic composition and content varied, our study showed that stable, consistent lots of MGSs can be prepared commercially, an important consideration for the use of MGS in clinical trials as a therapeutic or for consumer consumption as a preventive agent.

## Data Availability

All data are in the text, tables or figures found in the manuscript or the supplement.

## Supplementary Materials

The following supporting information can be downloaded at: https://www.mdpi.com/article/10.3390/antiox11112117/s1, Table S1: Retention times and mass to charge ratios of phenolic standards.

## References

[1] Bukhari S.N.A. (2022). Dietary Polyphenols as Therapeutic Intervention for Alzheimer’s Disease: A Mechanistic Insight. Antioxidants.

[2] Rudrapal M., Khairnar S.J., Khan J., Dukhyil A.B., Ansari M.A., Alomary M.N., Alshabrmi F.M., Palai S., Deb P.K., Devi R. (2022). Dietary Polyphenols and Their Role in Oxidative Stress-Induced Human Diseases: Insights Into Protective Effects, Antioxidant Potentials and Mechanism(s) of Action. Front. Pharmacol..

[3] Grosso G., Godos J., Currenti W., Micek A., Falzone L., Libra M., Giampieri F., Forbes-Hernández T.Y., Quiles J.L., Battino M. (2022). The Effect of Dietary Polyphenols on Vascular Health and Hypertension: Current Evidence and Mechanisms of Action. Nutrients.

[4] Sharma B.R., Jaiswal S., Ravindra P.V. (2022). Modulation of gut microbiota by bioactive compounds for prevention and management of type 2 diabetes. Biomed. Pharmacother..

[5] Arrigoni R., Ballini A., Santacroce L., Cantore S., Inchingolo A., Inchingolo F., Di Domenico M., Quagliuolo L., Boccellino M. (2022). Another Look at Dietary Polyphenols: Challenges in Cancer Prevention and Treatment. Curr. Med. Chem..

[6] Hoskin R.T., Xiong J., Lila M.A. (2019). Comparison of berry juice concentrates and pomaces and alternative plant proteins to produce spray dried protein-polyphenol food ingredients. Food Funct..

[7] Xia L., Xu C., Huang K., Lu J., Zhang Y. (2019). Evaluation of phenolic compounds, antioxidant and antiproliferative activities of 31 grape cultivars with different genotypes. J. Food Biochem..

[8] Sandhu A.K., Gu L. (2010). Antioxidant capacity, phenolic content, and profiling of phenolic compounds in the seeds, skin, and pulp of Vitis rotundifolia (Muscadine Grapes) As determined by HPLC-DAD-ESI-MS(n). J. Agric. Food Chem..

[9] Huang Z., Wang B., Eaves D.H., Shikany J.M., Pace R.D. (2009). Total phenolics and antioxidant capacity of indigenous vegetables in the southeast United States: Alabama Collaboration for Cardiovascular Equality Project. Int. J. Food Sci. Nutr..

[10] Paller C.J., Denmeade S.R., Carducci M.A. (2016). Challenges of conducting clinical trials of natural products to combat cancer. Clin. Adv. Hematol. Oncol..

[11] Atanasov A.G., Zotchev S.B., Dirsch V.M., Supuran C.T. (2021). Natural products in drug discovery: Advances and opportunities. Nat. Rev. Drug Discov..

[12] Wei Z., Luo J., Huang Y., Guo W., Zhang Y., Guan H., Xu C., Lu J. (2017). Profile of Polyphenol Compounds of Five Muscadine Grapes Cultivated in the United States and in Newly Adapted Locations in China. Int. J. Mol. Sci..

[13] You Q., Chen F., Sharp J.L., Wang X., You Y., Zhang C. (2012). High-performance liquid chromatography-mass spectrometry and evaporative light-scattering detector to compare phenolic profiles of muscadine grapes. J. Chromatogr. A.

[14] You Q., Chen F., Wang X., Sharp J.L., You Y. (2012). Analysis of phenolic composition of Noble muscadine (*Vitis rotundifolia*) by HPLC-MS and the relationship to its antioxidant capacity. J. Food Sci..

[15] Villani T.S., Reichert W., Ferruzzi M.G., Pasinetti G.M., Simon J.E., Wu Q. (2015). Chemical investigation of commercial grape seed derived products to assess quality and detect adulteration. Food Chem..

[16] Paller C.J., Rudek M.A., Zhou X.C., Wagner W.D., Hudson T.S., Anders N., Hammers H.J., Dowling D., King S., Antonarakis E.S. (2015). A phase I study of muscadine grape skin extract in men with biochemically recurrent prostate cancer: Safety, tolerability, and dose determination. Prostate.

[17] Paller C.J., Zhou X.C., Heath E.I., Taplin M.E., Mayer T., Stein M.N., Bubley G.J., Pili R., Hudson T., Kakarla R. (2018). Muscadine Grape Skin Extract (MPX) in Men with Biochemically Recurrent Prostate Cancer: A Randomized, Multicenter, Placebo-Controlled Clinical Trial. Clin. Cancer Res..

[18] Bitting R.L., Tooze J.A., Isom S., Petty W.J., Grant S.C., Desnoyers R.J., Thomas A., Thomas C.Y., Alistar A.T., Golden S.L. (2021). Phase I Study of Muscadine Grape Extract for Patients With Advanced Cancer. Am. J. Clin. Oncol..

[19] Singleton V. (1999). Colorimetry of total phenolics with phosphomolybdic-phosphotungstic acid reagents. Meth. Enzymol..

[20] Touyz R.M., Rios F.J., Alves-Lopes R., Neves K.B., Camargo L.L., Montezano A.C. (2020). Oxidative Stress: A Unifying Paradigm in Hypertension. Can. J. Cardiol..

[21] Shah A.K., Bhullar S.K., Elimban V., Dhalla N.S. (2021). Oxidative Stress as A Mechanism for Functional Alterations in Cardiac Hypertrophy and Heart Failure. Antioxidants.

[22] Tallant E.A., Ferrario C.M., Gallagher P.E. (2005). Angiotensin-(1-7) inhibits growth of cardiac myocytes through activation of the mas receptor. Am. J. Physiol. Heart Circ. Physiol..

[23] Collard M., Gallagher P.E., Tallant E.A. (2020). A Polyphenol-Rich Extract From Muscadine Grapes Inhibits Triple-Negative Breast Tumor Growth. Integr. Cancer Ther..

[24] Gallagher P.E., Ferrario C.M., Tallant E.A. (2008). Regulation of ACE2 in cardiac myocytes and fibroblasts. Am. J. Physiol.-Heart Circ. Physiol..

[25] Alfei S., Marengo B., Zuccari G. (2020). Oxidative Stress, Antioxidant Capabilities, and Bioavailability: Ellagic Acid or Urolithins?. Antioxidants.

[26] Sharifi-Rad J., Quispe C., Castillo C.M.S., Caroca R., Lazo-Vélez M.A., Antonyak H., Polishchuk A., Lysiuk R., Oliinyk P., De Masi L. (2022). Ellagic Acid: A Review on Its Natural Sources, Chemical Stability, and Therapeutic Potential. Oxid. Med. Cell. Longev..

[27] Xu Y., Tang G., Zhang C., Wang N., Feng Y. (2021). Gallic Acid and Diabetes Mellitus: Its Association with Oxidative Stress. Molecules.

[28] Tuli H.S., Mistry H., Kaur G., Aggarwal D., Garg V.K., Mittal S., Yerer M.B., Sak K., Khan M.A. (2022). Gallic Acid: A Dietary Polyphenol that Exhibits Anti-neoplastic Activities by Modulating Multiple Oncogenic Targets. Anticancer Agents Med. Chem..

[29] Jantan I., Haque M.A., Arshad L., Harikrishnan H., Septama A.W., Mohamed-Hussein Z.A. (2021). Dietary polyphenols suppress chronic inflammation by modulation of multiple inflammation-associated cell signaling pathways. J. Nutr. Biochem..

[30] Bai J., Zhang Y., Tang C., Hou Y., Ai X., Chen X., Wang X., Meng X. (2021). Gallic acid: Pharmacological activities and molecular mechanisms involved in inflammation-related diseases. Biomed. Pharmacother..

[31] Bao L., Cai X., Dai X., Ding Y., Jiang Y., Li Y., Zhang Z. (2014). Grape seed proanthocyanidin extracts ameliorate podocyte injury by activating peroxisome proliferator-activated receptor-γ coactivator 1α in low-dose streptozotocin-and high-carbohydrate/high-fat diet-induced diabetic rats. Food Funct..

[32] Bao L., Zhang Z., Dai X., Ding Y., Jiang Y., Li Y. (2015). Effects of grape seed proanthocyanidin extract on renal injury in type 2 diabetic rats. Mol. Med. Rep..

[33] Ding Y., Zhang Z., Dai X., Jiang Y., Bao L., Li Y. (2013). Grape seed proanthocyanidins ameliorate pancreatic beta-cell dysfunction and death in low-dose streptozotocin- and high-carbohydrate/high-fat diet-induced diabetic rats partially by regulating endoplasmic reticulum stress. Nutr. Metab..

[34] Ding Y., Dai X., Jiang Y., Zhang Z., Bao L., Li Y., Zhang F., Ma X., Cai X., Jing L. (2013). Grape seed proanthocyanidin extracts alleviate oxidative stress and ER stress in skeletal muscle of low-dose streptozotocin- and high-carbohydrate/high-fat diet-induced diabetic rats. Mol. Nutr. Food Res..

[35] Ho L., Chen L.H., Wang J., Zhao W., Talcott S.T., Ono K., Teplow D., Humala N., Cheng A., Percival S.S. (2009). Heterogeneity in red wine polyphenolic contents differentially influences Alzheimer’s disease-type neuropathology and cognitive deterioration. J. Alzheimers Dis..

[36] Wang J., Ho L., Zhao W., Ono K., Rosensweig C., Chen L., Humala N., Teplow D.B., Pasinetti G.M. (2008). Grape-derived polyphenolics prevent Abeta oligomerization and attenuate cognitive deterioration in a mouse model of Alzheimer’s disease. J. Neurosci..

[37] Ferruzzi M.G., Lobo J.K., Janle E.M., Cooper B., Simon J.E., Wu Q.L., Welch C., Ho L., Weaver C., Pasinetti G.M. (2009). Bioavailability of gallic acid and catechins from grape seed polyphenol extract is improved by repeated dosing in rats: Implications for treatment in Alzheimer’s disease. J. Alzheimers Dis..

[38] Hid E.J., Mosele J.I., Prince P.D., Fraga C.G., Galleano M. (2022). (-)-Epicatechin and cardiometabolic risk factors: A focus on potential mechanisms of action. Pflug. Arch..

[39] Oteiza P.I., Fraga C.G., Galleano M. (2021). Linking biomarkers of oxidative stress and disease with flavonoid consumption: From experimental models to humans. Redox Biol..

[40] Xu C., Yagiz Y., Zhao L., Simonne A., Lu J., Marshall M.R. (2017). Fruit quality, nutraceutical and antimicrobial properties of 58 muscadine grape varieties (*Vitis rotundifolia* Michx.) grown in United States. Food Chem..

[41] Ríos J.L., Giner R.M., Marín M., Recio M.C. (2018). A Pharmacological Update of Ellagic Acid. Planta Med..

[42] Tomás-Barberán F.A., González-Sarrías A., García-Villalba R., Núñez-Sánchez M.A., Selma M.V., García-Conesa M.T., Espín J.C. (2017). Urolithins, the rescue of “old” metabolites to understand a “new” concept: Metabotypes as a nexus among phenolic metabolism, microbiota dysbiosis, and host health status. Mol. Nutr. Food Res..

[43] García-Niño W.R., Ibarra-Lara L., Cuevas-Magaña M.Y., Sánchez-Mendoza A., Armada E. (2022). Protective activities of ellagic acid and urolithins against kidney toxicity of environmental pollutants: A review. Environ. Toxicol. Pharmacol..

[44] Chappell M.C., Pirro N., Melo A.C., Tallant E.A., Gallagher P. (2020). The microbiome product Urolithin A abrogates TGF-β-EGFR-PAI-1 pathway in NRK-52e renal epithelial cells. J Cell Signal..

