# Supplementary material for: Targeted UHPLC-MS Analysis Reveals Disparate Polyphenol Composition and Concentration in Muscadine Grape Supplements with Proportional Antioxidant Activity
Source: Antioxidants (Basel). 2022 Oct 27;11(11):2117. doi: 10.3390/antiox11112117 (PMC9686579; doi:10.3390/antiox11112117)
Supplement: Supplementary file 1 [file antioxidants-11-02117-s001.zip › antioxidants-1920728-supplementary/antioxidants-1920728-supplementary.pdf]

Table S1. Retention times and mass to charge ratios of phenolic standards

Phenolic Compounds	Molecular Formula	Retention (mins)	m/z (actual)	m/z (theoretical)	Error (ppm)
Gallic	$C_7H_6O_5$	0.826	169.0129	169.0143	-8.2833
Gallocatechin	$C_{15}H_{14}O_7$	0.967	305.0442	305.0667	-73.7544
Epigallocatechin	$C_{15}H_{14}O_7$	1.377	305.0398	305.0667	-88.1774
Isotopic Catechin	$C_{15}H_{14}O_6$	1.515	292.0903	292.0818	29.1014
Catechin	$C_{15}H_{14}O_6$	1.525	289.0435	289.0718	-101.3589
Caffeic	$C_9H_8O_4$	1.814	179. 0337	179.0350	-7.2612
Procyanidin B2	$C_{30}H_{26}O_{12}$	1.899	577.1368	577.1352	2.7723
Epicatechin	$C_{15}H_{14}O_6$	1.997	289.0723	289.0718	-97.7681
Gallocatechin gallate	$C_{22}H_{18}O_{11}$	2.109	457.0801	457.0776	12.033
p-Coumaric	$C_9H_8O_3$	2.345	163.0376	163.0401	-15.3337
Catechin gallate	$C_{22}H_{18}O_{10}$	2.505	441.0846	441.0827	4.3078
Ferrulic	$C_{10}H_{10}O_4$	2.704	193.0438	193.0501	-32.6340
Ellagic	$C_{14}H_6O_8$	2.779	301.0019	300.9990	9.6346
Myricetin	$C_{15}H_{10}O_8$	2.938	317.0272	317.0303	-9.7783
Resveratrol	$C_{14}H_{12}O_3$	2.923	227.0418	227.0714	-119.3457
Quercetin	$C_{15}H_{10}O_7$	3.309	301.0297	301.0348	-16.9416
Kaempferol	$C_{15}H_{10}O_6$	3.578	285.0369	285.0405	-12.6298
